# Network Specificity in Predicting Childhood Trauma Characteristics Using Effective Connectivity

**DOI:** 10.31083/AP43988

**Published:** 2025-06-18

**Authors:** Shufei Zhang, Wei Zheng, Zezhi Li, Huawang Wu

**Affiliations:** ^1^Institute of Neuroscience and Medicine, Brain and Behaviour (INM-7), Research Centre Jülich, 52425 Jülich, Germany; ^2^Institute for Systems Neuroscience, Medical Faculty, Heinrich-Heine University Düsseldorf, 40225 Düsseldorf, Germany; ^3^The Affiliated Brain Hospital, Guangzhou Medical University, 510370 Guangzhou, Guangdong, China; ^4^Key Laboratory of Neurogenetics and Channelopathies of Guangdong Province and The Ministry of Education of China, Guangzhou Medical University, 510180 Guangzhou, Guangdong, China

**Keywords:** effective connectivity, childhood maltreatment, regression dynamic causal modeling, default mode network, feature selection

## Abstract

**Background:**

Childhood maltreatment (CM) has become one of the leading psychological stressors, adversely impacting brain development during adolescence and into adulthood. Although previous studies have extensively explored functional connectivity associated with CM, the dynamic interaction of brain effective connectivity (EC) is not well documented.

**Methods:**

Resting-state functional magnetic resonance imaging data were collected from 215 adults with an assessment using the Childhood Trauma Questionnaire (CTQ). Whole-brain EC was estimated by regression dynamic causal modeling and subsequently down-resampled into seven networks. To predict CTQ total scores, repeated cross-validated ridge-regularized linear regression was employed, with whole-brain and network-specific EC features selected at thresholds of 5% of the strongest positive and negative correlations between EC and scores, as well as 10% and 20% thresholds. Additionally, a least absolute shrinkage and selection operator (LASSO)-regularized linear regression model was utilized as validation analysis.

**Results:**

Our findings revealed that whole-brain EC showed a marginal association with predicting CTQ total scores, and EC within the default mode network (DMN) significantly predicted these scores. EC features from other networks did not yield significant predictive results. Notably, across varying feature selection thresholds, DMN features consistently demonstrated significant predictive power, comparable to results from LASSO-regularized predictions.

**Conclusions:**

These findings suggested that brain EC can capture individual differences in CM severity, with the DMN potentially serving as an important predictor related to CM.

## Main points

1. Effective connectivity (EC) can capture individual differences in childhood maltreatment severity.

2. The EC features within the default mode network (DMN) showed the highest prediction correlation over other networks.

3. The DMN may serve as an important predictor associated with childhood maltreatment.

## 1. Introduction

Childhood maltreatment (CM), which encompasses different forms of maltreatment such as neglect and physical, sexual, and emotional abuse, has become one of the leading psychological stressors, negatively affecting brain development during adolescence and into adulthood [1]. Childhood experiences of adversity are significantly related to the first onset of psychiatric disorders with social-affective disturbances [2]. Individuals with CM may have a higher risk of developing bipolar disorder, depression, substance abuse, and suicidal behaviors [3, 4, 5]. Thus, understanding the neural substrate influenced by CM may be essential for creating preventive measures for individuals and offering therapeutic insights for treating psychiatric disorders.

Brain connectivity inferred from resting-state functional magnetic resonance imaging (rs-fMRI) has been widely used to explore the brain-behavior relationship. Functional connectivity (FC), one of the most commonly used connectivity measures, is defined by the pairwise correlation between distant brain regions and is considered a potential biomarker for human brain development and psychological processes [6]. A recent review [7] demonstrated that altered FC in brain regions such as the insula, amygdala, hippocampus, cingulate cortex, and prefrontal cortex is associated with exposure to individual maltreatment. Furthermore, accumulating evidence [8, 9] indicates that FC alterations within networks of the default mode network (DMN) and the salience network (SN) contribute to the pathophysiology of mental health disorders and social-affective functioning in individuals with maltreatment exposures. However, these studies [7, 8, 9] primarily focused on bidirectional connections and overlooked the dynamic influence that one region has on another [10].

Conversely, effective connectivity (EC), estimated by dynamic causal modeling (DCM), measures directional causal influences among brain regions [11]. This complements FC by a mechanistic explanation of the causal interactions through the modeling of directional causal influences [10] and may offer higher sensitivity than FC in psychopathology [12]. Although EC research related to the CM topic is sparse, a recent study [13] found that CM experiences impaired the medial prefrontal cortex’s ability to inhibit the amygdala during emotional processing, suggesting that EC could serve as a potential biomarker for psychiatric disorders. Additionally, unlike FC studies, many EC studies are driven by hypotheses and have typically limited their analyses to fewer than 10 nodes [13, 14], exploring the information flow directionality among specific network nodes. Additionally, due to the computational limitations, inferring EC at a whole-brain or large-scale level is challenging [15], leading to a restriction in the analysis of whole-brain and large-scale EC patterns. As emerging evidence suggests that CM is related to altered complex and distributed network architectures [16], examining whole-brain EC allows us to capture dynamic influences across widespread regions. This approach provides a more comprehensive view of how maltreatment experiences may disrupt functional network coupling, enhancing our understanding of CM’s neurobiological impact on broad connectivity dynamics.

In this study, we aim to evaluate whole-brain EC in individuals with CM and assess its potential for predicting CM severity. We measured individual CM severity using the Childhood Trauma Questionnaire (CTQ) Short Form [17] and estimated EC for all participants using regression DCM (rDCM) [14], which is a new variant of DCM. We then applied a cross-validated linear regression model to examine the relationship between whole-brain or network-specific EC profiles and CM severity.

## 2. Methods

### 2.1 Subjects

We recruited 215 healthy adult participants from Guangzhou Medical University and the surrounding community between July, 2013 and August, 2019. To ensure eligibility, we administered the Structured Clinical Interview for the Diagnostic and Statistical Manual of Mental Disorders–IV Edition (DSM- IV) Non-Patient Edition to all participants, confirming that they had no history of Axis I disorders. Additionally, we excluded anyone with a family history of psychiatric illness among first- to third-degree biological relatives, as well as individuals with a history of seizures, head trauma, significant surgeries or medical conditions, substance abuse or dependence, or contraindications for magnetic resonance imaging (MRI). We excluded nine subjects due to quality control issues of head motion and spatial normalization, leaving 206 subjects eligible for subsequent analysis (Table 1).

**Table 1.** **Demographic information and CTQ scores**.

Variable		Subjects (N = 206)
Sex (M/F)		91/115
Age (years)		25.3 ± 6.2 (18∼44)
Education (years)		14.0 ± 2.6 (4∼22)
Education Level		
	0∼6 years	1.5%
	6∼9 years	4.9%
	9∼12 years	22.3%
	>12 years	71.4%
CTQ Total Score		34.7 ± 7.8 (25∼61)
Emotional Abuse		6.6 ± 2.1 (5∼17)
Physical Abuse		5.8 ± 1.5 (5∼13)
Sexual Abuse		5.3 ± 0.8 (5∼11)
Emotional Neglect		9.6 ± 4.0 (5∼24)
Physical Neglect		7.3 ± 2.5 (5∼16)

Note: The percentage of education levels may not sum to 100% due to rounding. Values are presented as means ± standard deviations. Ranges for each variable are indicated in parentheses. Abbreviations: CTQ, Childhood Trauma Questionnaire; M/F, male/female.

All participants were right-handed and provided written informed consent before their involvement in the study. The study protocol received ethical approval from the Institute Research Board of the Affiliated Brain Hospital, Guangzhou Medical University.

### 2.2 Childhood Maltreatment Assessment

Before MRI scanning sessions, we conducted a thorough assessment of CM severity for all participants using the Chinese version of the CTQ Short Form [17, 18]. This self-report scale comprises 28 items, encompassing five distinct dimensions: emotional abuse, physical abuse, sexual abuse, emotional neglect, and physical neglect, and each item is scored from 1 (“never”) to 5 (“very often”). This scale has been widely applied in clinical and non-clinical populations [18], and its Chinese version has shown strong reliability and validity [17]. A detailed assessment of the CTQ scale and subscales for all qualified subjects (N = 206) can be seen in Table 1.

### 2.3 Imaging Protocols 

All MRI datasets for all participants were acquired utilizing a 3T Philips Achieva X-series MRI scanner, equipped with an eight-channel phased-array head coil in the Affiliated Brain Hospital, Guangzhou Medical University.

The resting-state fMRI datasets were collected using a gradient-echo echo-planar imaging sequence, with parameters as follows: repetition time (TR) = 2000 ms, echo time (TE) = 30 ms, flip angle = 90°, field of view (FOV) = 220 mm × 220 mm, and acquisition matrix = 64 × 64. The scan consisted of 33 transverse interleaved slices, each with a thickness of 4 mm and a gap of 0.6 mm. Throughout the scanning process, participants were instructed to remain at rest with their eyes closed, and none of them reported falling asleep upon being queried immediately after the scan.

A T1-weighted 3D turbo field-echo sequence was employed to acquire the structural datasets, with parameters as follows: TR = 8.2 ms, TE = 3.7 ms, inversion time = 1100 ms, flip angle = 7°, FOV = 256 mm × 256 mm, acquisition matrix = 256 × 256, and voxel size = 1 × 1 × 1 mm^3^. The scan encompassed continuous 188 sagittal slices covering the brain.

### 2.4 Imaging Preprocessing

The resting-state fMRI images were preprocessed by the Data Processing and Analysis for Brain Imaging (DPABI) pipeline (http://rfmri.org/dpabi), as implemented in the Matlab (v 2016a, Mathworks, Natick, MA, USA) platform.

The preprocessing pipeline included the following steps: (1) removal of the first 10 volumes; (2) slice-timing and head-motion correction; (3) co-registration of functional images to structural space; (4) regression of whole-brain, cerebrospinal fluid, and white matter signals, as well as linear trends and Friston-24 head-motion parameters; (5) non-linear normalization to Montreal Neurological Institute (MNI) space; (6) smoothing with a 5-mm full-width-at-half-maximum; and (7) temporal filtering within a frequency range of 0.01 to 0.1 Hz.

After preprocessing, nine participants were excluded due to quality control issues of the head motion and spatial normalization, and 206 participants were kept for further analyses.

**Fig. 1.**
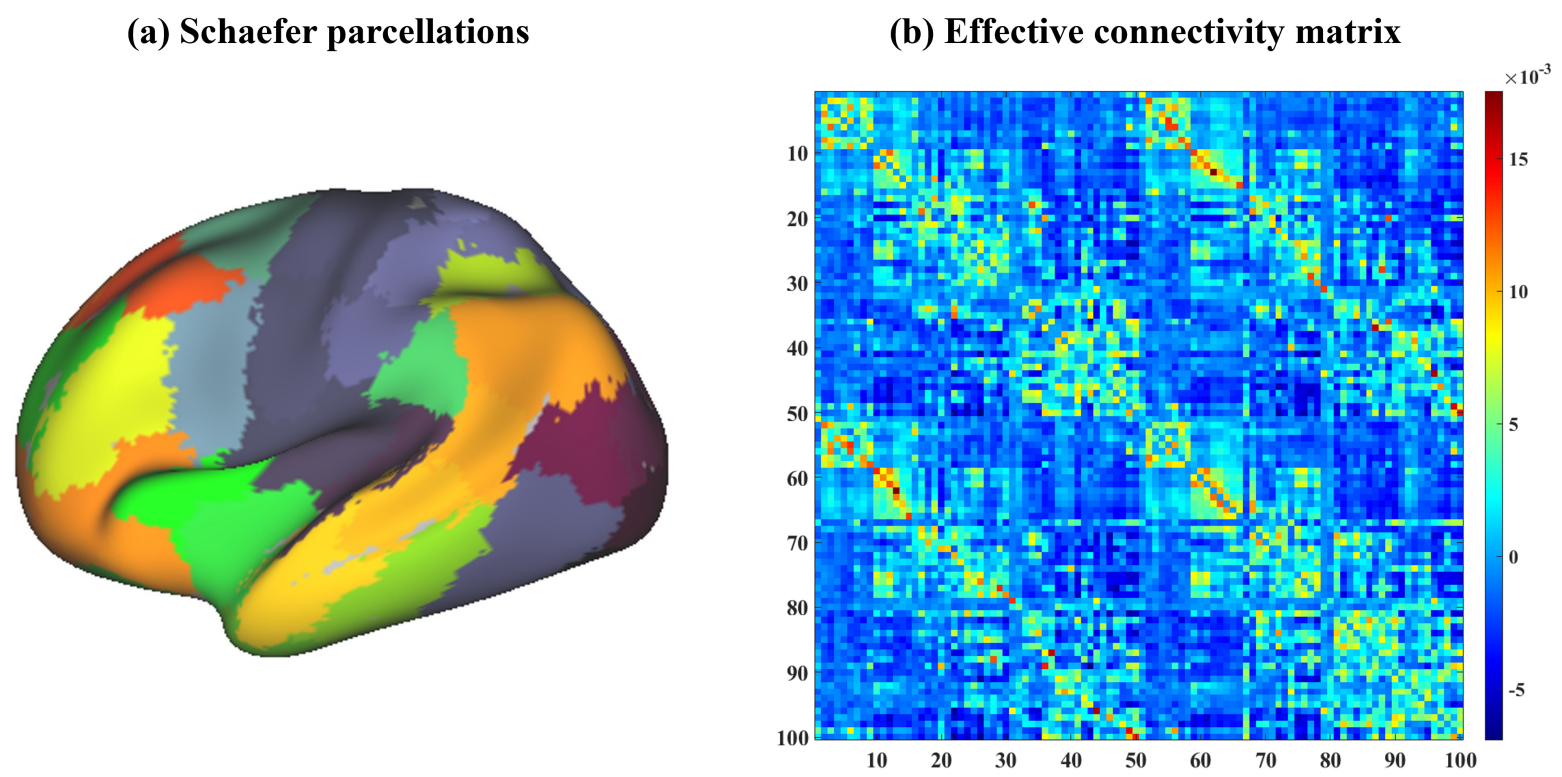
**The Schaefer atlas and effective connectivity (EC) matrix averaged across all participants**. (a) shows the utilized Schaefer atlas (100 parcels) used for extracting individual timeseries and the color indicates the different parcellations. (b) illustrates averaged EC across subjects and the color bar indicates the EC magnitude.

For the estimation of individual EC, we applied the Schaefer atlas [19] (100 parcels, Fig. 1a) to cover the cortex and extracted the first eigenvariate for each region from the preprocessed functional images using fslmeants/FSL. This eigenvariate served as the time series data, which was then utilized in the calculation of EC.

### 2.5 Effective Connectivity Estimation

As a recently developed variant of DCM, rDCM was utilized to estimate individual whole-brain EC efficiently [14]. This method transforms conventional linear DCM into the frequency domain, making it a special case of the Bayesian linear regression model [14]. Using this approach, we specified a full-connection model to estimate EC, where a 100-by-100 matrix was built for each subject (Fig. 1b). Additionally, to examine the prediction performance from separate networks, we down-resampled the whole-brain EC matrix into seven networks including the visual network (Vis), somatosensory network (SMN), dorsal attentional network (DAN), ventral attentional network (VAN), limbic network (Lim), fronto-parietal network (FPN), and DMN. This down-sampling grouped parcels based on the Schaefer atlas [19, 20], aligning each of the 100 parcels with one of seven networks. We extracted within-network connectivity for each network by selecting matrix entries where nodes shared the same network label. This approach enabled a targeted comparison of whole-brain and network-specific EC predictions.

### 2.6 Machine Learning

To explore the relationship between whole-brain and network-specific EC and CM severity, we employed a ridge-regularized linear regression model within a 10-fold cross-validation scheme, where EC was specified as features and the total scores summing from all CTQ subscales were used for targets. The cross-validation process involved splitting samples into training and testing sets. In the training set, EC features were correlated with CTQ total scores, and the top 5% of features were extracted based on the highest positive and negative correlations, respectively. A ridge-regularized linear regression model was then trained on the training set, which was subsequently used to predict scores in the testing set, calculating the correlations between predicted and actual scores. An average prediction correlation was calculated across all cross-validation folds.

To improve the stability of prediction performance, we repeated this prediction process 100 times and obtained an average prediction correlation across all iterations for each prediction case. To assess the statistical performance of the prediction model with the feature selection threshold of 5%, we applied a label-shuffled permutation (n = 500 times) test, where CTQ total scores were randomly shuffled before each cross-validation loop. The permutation process serves to evaluate the robustness of our model by generating a null distribution of prediction correlations. By comparing the empirical prediction performance against this null distribution, we can determine the statistical significance of our observed results with a threshold of *p *= 0.05. Given that predictions were conducted for the whole brain and each of the seven networks, we additionally applied a Bonferroni-corrected threshold of *p* = 0.0063 (0.05/8) for multiple comparisons to the uncorrected *p*-values obtained from each permutation test. This provides both uncorrected and corrected interpretations, offering a more comprehensive view of our findings.

Considering that feature selection thresholds may impact prediction performance, we repeated our prediction processes with the feature selection conditions of 10% and 20%, which enabled us to confirm the consistency of prediction performance patterns among whole-brain and network-specific predictions. Additionally, to verify our findings, we employed a least absolute shrinkage and selection operator (LASSO)-regularized linear regression model and compared its results with those of the ridge-regularized model at a 5% feature selection threshold.

## 3. Results

### 3.1 Demographic Information and CTQ Scores

Table 1 summarizes the demographic characteristics and CTQ scores for the 206 participants included in the study after imaging preprocessing. The sample comprised 91 males and 115 females, with an average age of 25.3 years. Participants had an average of 14 years of education. The mean CTQ total score was 34.7, with subscale averages as follows: emotional abuse, 6.6; physical abuse, 5.8; sexual abuse, 5.3; emotional neglect, 9.6; and physical neglect, 7.3.

### 3.2 Prediction Results

In this study, we examined the prediction correlation between CTQ total scores and EC features selected from the whole brain and separated networks using a ridge-regularized linear regression model, where a feature selection threshold of 5% was applied. Then, to improve the robustness and consistency of the above predictions, we also employed 10% and 20% feature selection thresholds to examine whole-brain and network-specific EC predictions. Additionally, a LASSO-regularized model was employed to validate the prediction results.

**Fig. 2.**
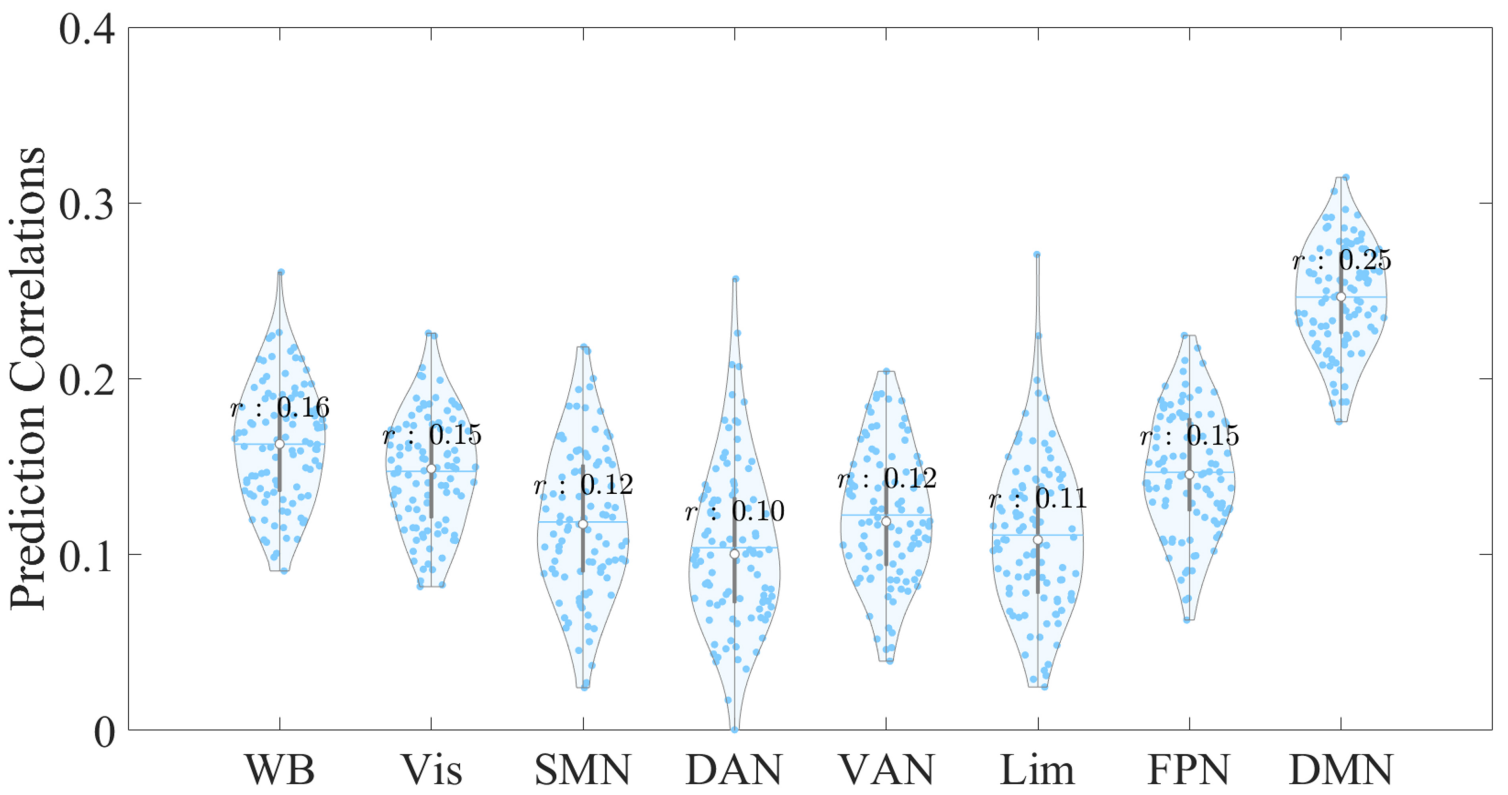
**Violin plot showing the distribution of prediction correlations for whole-brain and network-specific EC features derived from 100 repetitions with a 5% feature selection threshold**. The x-axis represents the EC feature sources used to predict the CTQ total scores, while the y-axis indicates the correlation between the empirical and predicted scores. Each dot represents an individual prediction correlation. Abbreviations: WB, whole brain; Vis, visual network; SMN, somatosensory network; DAN, dorsal attentional network; VAN, ventral attentional network; Lim, limbic network; FPN, fronto-parietal network; DMN, default mode network.

When considering a feature selection threshold of 5% (Fig. 2 and Table 2), we found that whole-brain EC features marginally predicted CTQ total scores, with a correlation coefficient of *r *= 0.16. However, when predictions were examined at the network-specific level, only EC features from the DMN successfully predicted CTQ total scores, yielding a stronger correlation of *r* = 0.25 (*p* < 0.05). In contrast, EC features from other networks, including the Vis, SMN, DAN, VAN, Lim, and FPN, failed to be statistically significant in prediction (*p* > 0.05). Their prediction correlations ranged from 0.1 to 0.15. Notably, EC features from the DMN outperformed those from all other cases in predicting CTQ total scores, exhibiting the highest prediction correlation compared with both the whole-brain and other network-specific predictions. However, when including a multiple-comparison corrected threshold of *p* = 0.0063 (Bonferroni corrected), none of them were statistically significant.

**Table 2.** **Correlation coefficients (*r*) for predicting CTQ total scores using WB and network-specific EC features at a 5% feature selection threshold**.

Prediction Correlation	EC feature							
	WB	Vis	SMN	DAN	VAN	Lim	FPN	DMN
*r*	0.16	0.15	0.12	0.10	0.12	0.11	0.15	0.25
*p*	0.05	0.33	0.59	0.67	0.58	0.68	0.41	0.02 *

Note: An asterisk (*) indicates statistical significance with a *p*-value below the threshold for significance (*p* < 0.05) based on permutation testing.

### 3.3 Feature Selection Thresholds Analysis

To examine the impact of feature selection thresholds on prediction correlations for CTQ total scores, we varied the feature selection thresholds from 5% to 10% and 20% (Table 3). We observed that the significance level of whole-brain EC prediction did not vary across different thresholds, with no significant predictions for CTQ total scores at the 10% or 20% thresholds (*p* > 0.05). In contrast, EC features from the DMN remained statistically significant (*p* < 0.05) even after applying a multiple-comparison correction (*p* < 0.0063) with these thresholds. Importantly, varying the feature selection thresholds did not affect the statistical significance (*p* > 0.05) of EC features in the other networks.

**Table 3.** **Correlation coefficients (*r*) for predicting CTQ total scores using WB and network-specific EC features at 10% and 20% feature selection thresholds**.

Features		WB	Vis	SMN	DAN	VAN	Lim	FPN	DMN
10%	*r*	0.15	0.16	0.12	0.12	0.11	0.12	0.17	0.24
	*p*	0.07	0.30	0.56	0.49	0.66	0.67	0.27	0.00 **
20%	*r*	0.15	0.15	0.12	0.12	0.11	0.12	0.17	0.23
	*p*	0.08	0.12	0.49	0.56	0.66	0.57	0.21	0.00 **

Note: Double asterisks (**) indicate statistical significance based on the permutation test with a *p*-value below the Bonferroni-corrected threshold of *p* = 0.0063 for multiple comparisons.

### 3.4 Validity Analysis

**Fig. 3.**
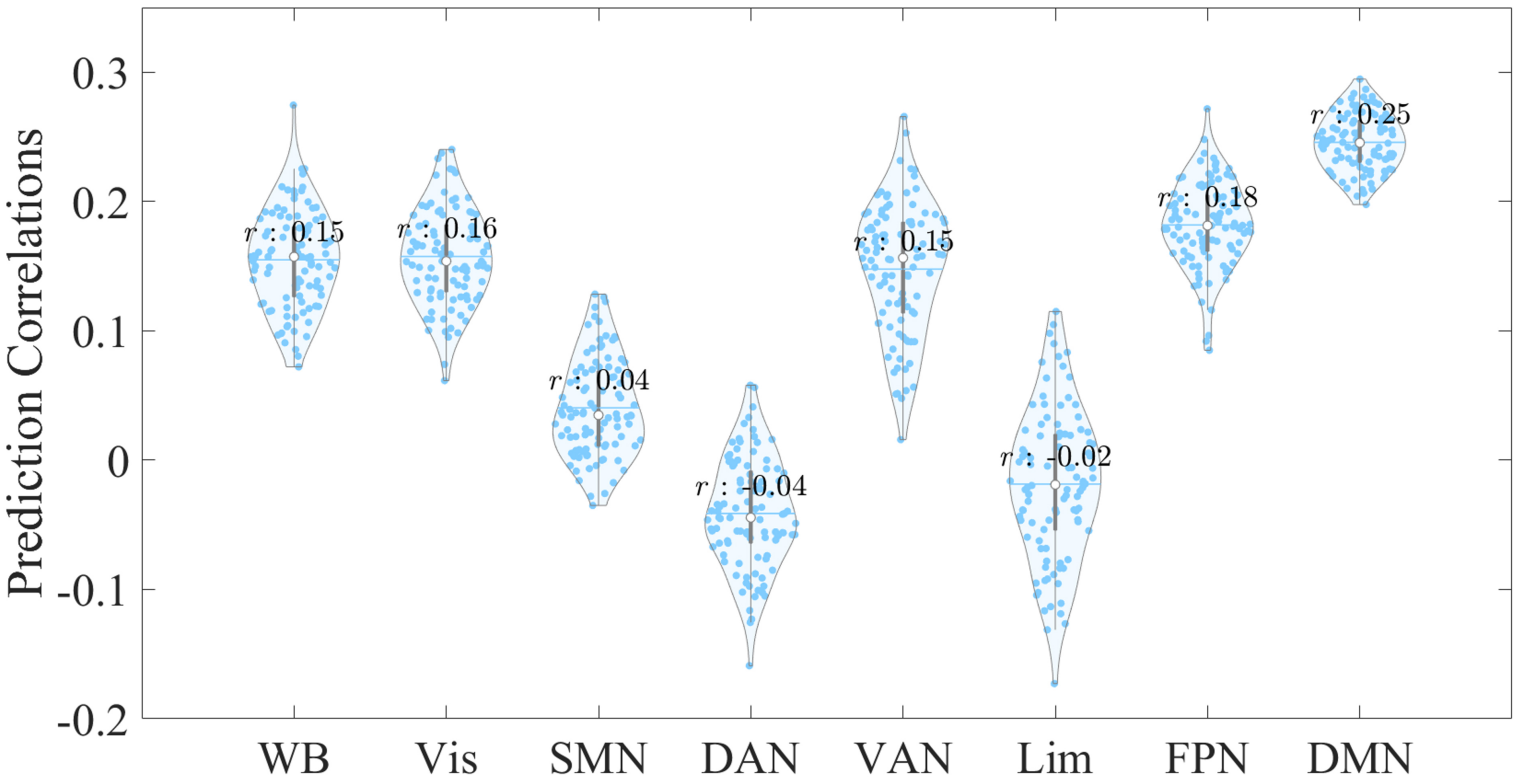
**Violin plot displaying the prediction distributions for whole-brain and network-specific EC features, derived from 100 repetitions at a 5% feature selection threshold using the LASSO-regularized linear regression model**. The x-axis represents the EC feature sources used to predict CTQ total scores, while the y-axis shows the correlation between empirical and predicted CTQ total scores. Abbreviations: LASSO, least absolute shrinkage and selection operator.

To validate our prediction results obtained from the ridge-regularized linear regression model, we then examined the prediction correlations using a LASSO-regularized model (Fig. 3). Our findings were consistent with those of the ridge-regularized model, and EC features within the DMN demonstrated the highest prediction correlation compared with other networks.

## 4. Discussion

To our knowledge, this study is the first to investigate large-scale network EC for predicting CM severity, employing whole-brain and network-specific EC to predict individual CM severity using a ridge-regularized linear regression model. Our results suggested that (*i*) EC features from the DMN show the best prediction performance over other prediction cases and (*ii*) varying feature selection thresholds may not evidently impact the statistical significance of predictions. Notably, the DMN’s predictive validity was further supported by the LASSO-regularized linear regression model. This suggested that the EC features of the DMN may play a crucial role in capturing individual differences in CM severity, which highlighted the potential of DMN connectivity as a more reliable predictor of clinical outcomes compared with connectivity of other large-scale networks.

### 4.1 Effective Connectivity Prediction

The present study has shown that EC was predictive of individual differences in CM severity. This aligns with previous literature reporting that the EC feature can classify clinical patients from healthy controls well and predict treatment outcomes and behavioral domains [21, 22, 23]. This supports the utility of EC as a promising feature in understanding and predicting clinical and behavioral variations.

Our study further demonstrated that EC features within the DMN were superior to those within other networks. The DMN structure primarily includes regions of the medial prefrontal cortex, posterior cingulate cortex, precuneus, and lateral parietal cortex [24]. This network is known to be engaged in internally-focused mental processes such as self-reflection, mind wandering, daydreaming, autobiographical memory retrieval, and future planning [25, 26, 27]. Aberrant activities in the DMN may contribute to multiple dysfunctional psychological processes in self-referential thinking and working memory, and attentional impairments [28, 29]. Given that the DMN is crucial in self-referential cognitive functions, it is of great importance in the study of CM [30]. Altered connectivity of the DMN is linked to various psychopathological symptoms in transdiagnostic samples including post-trauma stress disorder (PTSD), major depressive disorder (MDD), and schizophrenia [30, 31, 32]. In schizophrenia, the experiences of CM were associated with connectivity variations in the DMN [33]. In MDD, the altered functional coupling within the DMN may contribute to developing depression in individuals with CM [34]. In PTSD, reduced FC within the DMN may lead to self-perception disturbance and link traumatic experiences to the sense of self [35]. Accumulating evidence shows that traumatic exposure adversely impacts DMN connectivity, which in turn leads to psychopathological symptoms associated with maladaptive self-referential processes [30, 36]. Additionally, previous reviews have highlighted the DMN as a network of particular interest in non-clinical individuals exposed to CM [7, 36, 37]. Numerous studies [38, 39, 40, 41, 42] have documented altered connectivity within the DMN, as well as between the DMN and other networks, in individuals with CM compared with those without. For instance, Lu *et al*. [38] observed abnormal FC within the DMN and in its connections with the cerebellum and insula in individuals with CM, while Zhao *et al*. [39] reported increased connectivity within the anterior DMN and reduced connectivity between the posterior DMN and other networks in non-clinical adults with CM. Moreover, DMN connectivity has demonstrated strong predictive contributions for CTQ scores in non-clinical individuals [43], underscoring the DMN’s role as a potential biomarker of trauma. Collectively, these findings, together with existing literature on early-life stress [36, 44, 45], suggested that alterations in DMN connectivity may serve as important neural correlates of early-life adversity. Although research regarding EC and CM is sparse, several studies utilized prior-defined regions of interest to explore EC between the amygdala and medial prefrontal cortex, reporting that traumatic exposures impact the inhibition of the amygdala by the medial prefrontal cortex [13, 46]. This may potentially reflect dysfunction in the DMN, contributing to maladaptive emotional responses impacted by traumatic exposures [46]. Consistent with these studies, our findings demonstrated that EC features within the DMN better predicted CM scores than those from other networks, suggesting that the DMN may play a crucial role in influencing CM effects on self-referential and emotional regulation.

### 4.2 Feature Selection Thresholds and Predictive Models

This study further evaluated the stability of our prediction findings by varying the feature selection thresholds. Although the *p*-values for the statistical significance of EC prediction were impacted by different feature selection thresholds, the significance of most prediction cases was little influenced. This aligns with previous literature [47, 48] suggesting that varying feature selection thresholds (e.g., stricter *p* values) in selecting correlation-based features may not significantly impact prediction. While those studies primarily used *p*-values rather than a fixed percentage of connectivity features, our validation analysis across different thresholds demonstrated relatively consistent and robust prediction results, particularly for DMN EC features, where the predictions remained stable across different thresholds. Furthermore, the prediction results obtained from the ridge-regularized model were comparable to those from the LASSO model, showing similar stability of EC features within the DMN.

## 5. Limitations

Several limitations should be addressed in future studies. First, a key limitation of our study is the relatively low CTQ total and subscale scores reported in Table 1, with a limited number of participants reporting experiences of sexual, physical, or emotional abuse. This distribution suggests that our findings may be more strongly influenced by neglect-related experiences. This outcome may be partly related to the characteristics of our sample, as higher educational levels among participants could potentially impact reporting patterns, with fewer cases of abuse reported relative to neglect. Additionally, the narrow range of subscale scores reduced their predictive power, leading us to focus on the CTQ total score. Future studies including more diverse samples could help examine the distinct impacts of different types of CM. Second, this study assessed the CM severity in healthy participants without measuring psychopathological traits or symptoms such as anxiety or depression. The following studies may incorporate these assessments to better understand their potential influence on CM severity in healthy participants. Third, while our study highlighted the strong predictive correlation of DMN-based EC features in capturing individual differences in CM severity, the choice of performance metrics may influence model comparisons. Future studies should explore more metrics to better understand the relative predictive roles of different networks. Ultimately, the Schaefer atlas may impact network definitions, as it assigns different numbers of parcels to various networks. This variation could influence the predictive power of networks, potentially affecting our findings related to CM severity. Future studies could benefit from exploring alternative atlases or parcellation schemes, which may lead to a more comprehensive assessment of how different networks relate to CM and other forms of childhood adversity.

## 6. Conclusions

In conclusion, our study suggests that EC can effectively capture individual differences in CM severity, particularly within the DMN, which demonstrated the highest prediction correlations. These findings highlight the DMN’s significant potential as a neuroimaging biomarker for the underlying mechanisms of CM.

## Data Availability

The code and data can be made available upon reasonable request.
